# A mild alkali treated jute fibre controlling the hydration behaviour of greener cement paste

**DOI:** 10.1038/srep07837

**Published:** 2015-01-16

**Authors:** Byung-Wan Jo, Sumit Chakraborty

**Affiliations:** 1 Department of Civil and Environmental engineering, Hanyang University, Seoul, South Korea, 133791

## Abstract

To reduce the antagonistic effect of jute fibre on the setting and hydration of jute reinforced cement, modified jute fibre reinforcement would be a unique approach. The present investigation deals with the effectiveness of mild alkali treated (0.5%) jute fibre on the setting and hydration behaviour of cement. Setting time measurement, hydration test and analytical characterizations of the hardened samples (viz., FTIR, XRD, DSC, TGA, and free lime estimation) were used to evaluate the effect of alkali treated jute fibre. From the hydration test, the time (t) required to reach maximum temperature for the hydration of control cement sample is estimated to be 860 min, whilst the time (t) is measured to be 1040 min for the hydration of a raw jute reinforced cement sample. However, the time (t) is estimated to be 1020 min for the hydration of an alkali treated jute reinforced cement sample. Additionally, from the analytical characterizations, it is determined that fibre-cement compatibility is increased and hydration delaying effect is minimized by using alkali treated jute fibre as fibre reinforcement. Based on the analyses, a model has been proposed to explain the setting and hydration behaviour of alkali treated jute fibre reinforced cement composite.

Sustainable, energy efficient and eco-friendly construction material is sought around the world^1^,^2^. To develop sustainable, green construction material, natural fibre reinforcement in a cement matrix is a feasible approach. Fibres from natural sources have the potential to be used as reinforcement in cement composite to overcome the inherent brittleness of the cementitious materials. Adequate research has been done to develop high-performance fibres like bamboo, sisal, coconut and coir^3^,^4^,^5^ reinforced cement composites. The natural fibres in the cement matrix impart adequate toughness and ductility to the cement composite. However, the fibre-cement compatibility and hydration delaying activity of such fibres must be taken into consideration, which could be ensured by effective modifications either to the fibre surface or to the matrix composite. The fibre surface treatment is particularly important to reduce the hydration delaying activity and to improve the fibre strength as well as composite strength by improving the fibre matrix compatibility.

Hydration of cement is a complex sequence of interactions occurring between water and chemical phases of the cement (viz., tricalcium silicate (3CaO·SiO_2_), dicalcium silicate (2CaO·SiO), tricalcium aluminate (3CaO·Al_2_O_3_) and tetra calcium aluminoferrite ((Ca_2_(Al,Fe)_2_O_5_))^6^,^7^,^8^. Calcium hydroxide and calcium silicate hydrate are produced predominantly as hydrated cement products^9^,^10^. However, the cement hydration reaction equilibrium as well as nucleation and growth of the hydrated cement product may be disturbed by the presence of foreign substances. In natural fibre reinforced cement composite, the natural fibre leaches various organic soluble sugars in the cement medium, which form a protective layer around the cement grains^11^. The protective layer of sugars prevents water from percolating to further hydrate the cement grain^12^,^13^. Consequently, the nucleation and growth of the hydrated product is delayed. Therefore, an immediate practical plan is required to establish the convalescence of the antagonistic effect of the natural fibre. Modifying the fibre may improve the fibre matrix compatibility as well as reduce the hydration delaying effect. Some special treatments such as silane grafting, hot-cold water extraction, and chemical soaking of the natural fibres are reported elsewhere to achieve targeted properties^14^,^15^,^16^. Moreover, the alkali treatment is a unique scheme to remove soluble sugars from the fibre which remain dispersed in the interfibrillar region of the natural fibre^17^. Due to the removal of the impurities from the interfibrillar region of the natural fibres, the fibrils become more capable of rearranging themselves along the direction of tensile deformation, which in turn leads to enhanced mechanical properties of the fibre^17^. Additionally, fibrillation of the fibre due to alkali treatment leads to an increase in the effective surface area, which in turn increases the availability of the effective surface area for bonding between the fibre and the matrix at the interface^17^,^18^. Therefore, an encouraging effect of the alkali treated natural fibre is an improvement in the performances (mechanical and hydration) of the natural fibre reinforced cement composites.

Limited research reports are available for the alkali treatment of natural fibre; however, to the best of our knowledge, the effectiveness of the alkali modified jute fibre on the setting and the hydration behaviour of cement has not yet been studied. In this context, we have studied the effectiveness of alkali treated jute fibre as concrete reinforcement with regard to the setting and hydration of cement. The alkali treatment of jute fibre is demonstrated to be very effective to control the setting and the hydration behaviour of the jute fibre reinforced cement.

## Results

In order to evaluate the effectiveness of the mild alkali treated jute fibre for controlling the cement hydration reaction, we estimate the hydration of the cement at the early age and in the hardened state, including setting time measurement, in conjunction with analytical analyses such as FTIR, XRD, DSC, TGA. Table 1 depicts the standard consistency, initial setting time and final setting time of the control, raw jute reinforced and alkali treated jute reinforced cement samples. The standard consistency of cement is the minimum quantity (%) of water required to obtain an adequate wet mixture of cement. In this study, the value of standard consistency of the control cement sample is estimated to be 35%, and is measured to be 36.5% for both of the cement samples, raw jute fibre reinforced cement sample and alkali treated jute reinforced cement sample, respectively (shown in Table 1). These results indicate that the demand of water is increased for raw jute and alkali treated jute cement samples in order to obtain an adequate wet mixture of cement. Additionally, the initial and final setting times of the control cement are 130 min and 170 min, respectively. The two setting times increase to 160 min (initial) and 205 min (final) for the raw jute reinforced cement sample. On the other hand, the setting times are substantially reduced to 154 min (initial) and 194 min (final) for the alkali treated jute cement sample. It is therefore indicative to recover the setting delaying effect of jute fibre by mild alkali treatment. Interestingly, from Table 1, we see that the difference between the final and initial setting times of raw jute fibre reinforced cement increases as compared to the control sample; but decreases when alkali treated jute fibres are used as fibre reinforcement. Raw jute fibre used for fibre reinforcement delays the setting of the cement, which is counterbalanced by the addition of the alkali treated jute fibre as fibre reinforcement.

### The hydration analysis demonstrates evidence of cement hydration

**Figure 1:**
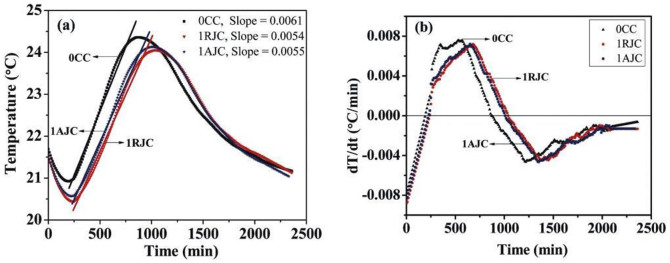
(a) Temperature vs. time curve and (b) temperature changing rate (dT/dt) vs. time (t) plot of control, raw jute, and alkali treated jute fibre reinforced cement samples.

The temperature vs. time and the rate of temperature change (dT/dt) vs. time (t) plots of control, raw jute, and alkali treated jute fibre reinforced cement samples are shown in Figs. 1(a) and 1(b), respectively. Table 2 represents the characteristic features of the temperature vs. time and rate of temperature change (dT/dt) vs. time (t) plots of the control cement paste, raw jute cement paste and alkali treated jute cement paste (shown in Figs. 1(a) and 1(b)). As indicated in Table 2, the maximum achievable temperature (T_max_) and the time required to reach the maximum temperature (t) are observed to be 24.37°C and 860 min, respectively, for the control cement; the values are estimated to be 24.05°C and 1040 min, respectively, for the raw jute reinforced cement sample. However, the above two values are predicted to be 24.07°C and 1020 min, respectively, for the alkali treated jute reinforced cement sample. It is likely that the hydration of cement is delayed by the addition of raw jute fibre. However, the addition of the alkali treated jute fibre as fibre reinforcement in the cement matrix reduces the delay in the hydration of cement.

Interestingly, from the temperature vs. time plot, it is apparent that the trend in the temperature from stage II to stage III is linear. The slope of the linear improvement in temperature is predicted to be 6 × 10^−3^ for control cement, which declines to 5.3 × 10^−3^, when raw jute fibre is used for fibre reinforcement in the cement system. The slope increases to be 5.4 × 10^−3^ with the addition of alkali treated jute fibre for fibre reinforcement in a cement system. This phenomenon suggests that the cement hydration reaction is delayed due to the use of raw jute fibre for fibre reinforcement. A delayed hydration reaction is counterbalanced by the use of alkali treated jute fibre for fibre reinforcement in a cement system.

Fig. 1(b) shows the rate of temperature change for control cement, raw jute cement and alkali treated jute cement samples from the beginning of hydration (stage I). The rate of temperature change increases gradually with hydration time until the trend levels off. A positive value of the rate of temperature change is observed immediately after tr_(zero)_). This seems to be due to the maximum dissolution of Ca^2+^ ions in the pore water solution at the beginning of the reaction. The dissolution of the Ca^2+^ ions decreases the exothermic reaction rate. In the dormant period, the rate of temperature change becomes zero and then accelerates. This may be due to the super saturation of Ca^2+^ ion in the pore solution and the crystallization process^19^. From the figure, we observe that the times required to reach zero and the maximum value of the rate of temperature change (tr_(zero)_) (tr_(max)_) of the control cement sample are 200 min and 570 min, respectively. The same values are estimated to be 240 min and 680 min, respectively, for the raw jute reinforced cement sample; nevertheless, these two values are measured to be 230 min and 650 min, respectively (Table 2), for the alkali treated jute reinforced cement sample. Therefore, we observe that the cement hydration reaction is significantly delayed by the addition of raw jute fibre for fibre reinforcement, which is recovered by the addition of alkali treated jute fibre for fibre reinforcement. The phenomenon is further clarified by the analytical characterizations (XRD, FTIR, DSC, and TG-DTG analyses, and the free lime estimation method).

### XRD analysis identifies the reactants and product phases of the hydrated cement

**Figure 2:**
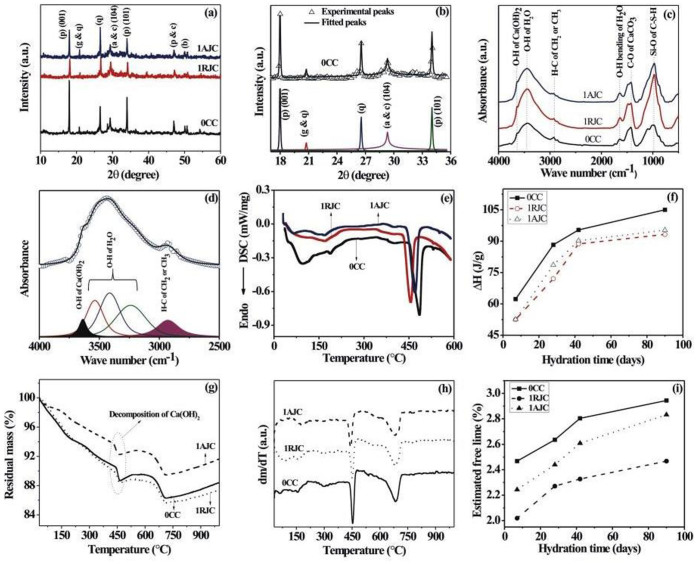
Analysis of the control, raw jute, and alkali treated jute reinforced cement samples hydrated for 28 days. (a) X-ray diffraction pattern of hydrated samples, (b) fitted XRD peaks of the control sample, (c) FTIR spectra of hydrated samples, (d) fitted FTIR peaks of control sample, (e) DSC of hydrated samples, (f) variation in enthalpy change with increasing hydration time, (g) TG of the hydrated cement sample and (h) DTG of the same, and (i) variation in the estimated free lime content as a function of the hydration time.

The X-ray diffraction patterns of the 28 days of hydrated control, raw jute, and alkali treated jute reinforced cement samples are shown in Fig. 2(a). The major components of Portland-pozzolana cement are alite (a) [tricalcium silicate C_3_S (Ca_3_SiO_5_)], belite (b) [dicalcium silicate C_2_S (Ca_2_SiO_4_)], tricalcium aluminate [C_3_A (Ca_3_Al_2_O_6_)], and tetracalcium aluminoferrite [C_4_AF (Ca_4_Al_n_Fe_2−n_O_7_,)]^20^,^21^. Additionally, it contains some amount of quartz (q) and gypsum (g) also. As represented in Fig. 2(a), the characteristic peak corresponding to the portlandite phase (p) appears at 2θ ~ 18°. In the X-ray diffractogram, the major reactant phase alite (a) is identified at 2θ ~ 29.4°. The integrated peak area ratio of the peaks corresponds to the portlandite (p) and the allite (a) phases and could therefore be treated as an index of the degree of hydration^12^. In this study, the X-ray diffraction patterns of the hydrated cement samples are fitted using commercial software (Peakfit 4.1, Jandel Scientific) to estimate the integrated peak area. Fig. 2(b) represents the fitted as well as deconvoluted XRD peaks of the control cement hydrated for 28 days. Chakraborty et al.^12^ reported the details of the fitting of the X-ray diffractogram.

The integrated peak area ratio of the peaks corresponds to the portlandite (p) and the alite (a) phases (A_p_/A_a_) of the control sample. After 28 days of hydration, it is estimated to be ~0.169, which is reduced to 0.105, when 1% jute is mixed with cement to produce a raw jute reinforced cement sample (Table 3). Nevertheless, the value of the integrated peak area ratio is predicted to be 0.125 for the alkali treated jute cement sample. This suggests that the less hydration occurs with the raw jute reinforced cement sample as compared to the control cement sample. However, the addition of the alkali modified jute fibre for fibre reinforcement increases the degree of cement hydration reaction compared to that of the raw jute reinforced cement sample. Additionally, from the X-ray diffraction analysis, we observe that the degree of hydration reaction of the control, raw jute, and alkali treated jute fibre reinforced cement samples increases gradually with increasing hydration time (as indicated in Table 3).

### FTIR analysis indicates the extent of the formation of hydrated cement product

Additionally, FTIR analysis of the cement samples allows a better understanding of the above effect. Fig. 2(c) shows the FTIR spectra of control, raw jute, and alkali treated jute fibre reinforced cement samples hydrated for 28 days. The hydrated cement samples show IR bands at 3638, 3441, 2928, 1649, 1422, 978 and 465 cm^−1^. The assignments of these absorption bands are already described by Chakraborty et al.^12^ and Ghosh^22^. The absorption band at ~3638 cm^−1^ appears to be due to the O-H stretching of the portlandite (Ca(OH)_2_) phase^23^. The band at ~2928 cm^−1^ appears to be due to the asymmetric stretching of the H-C bond of the organic moieties present in the cement sample^12^. This absorption band (~2928 cm^−1^) is considered as an internal standard^12^. The change in the relative intensity of the O-H stretching mode of the portlandite phase is estimated by fitting the FTIR bands using commercial software (Peakfit 4.1, Jandel Scientific). The typical fitting and the deconvoluted modes for the control specimen are shown in Fig. 2(d). A brief description of the deconvoluted fitting of the FTIR spectra was reported by Chakraborty et al.^12^. In this study, the ratio of the integrated peak areas of the peaks appears to be due to the O-H stretching of Ca(OH)_2_ mode and the asymmetric H-C stretching mode of the methylene and methyl group (A_O-H_/A_H-C_) for control cement, raw jute reinforced cement and alkali treated jute reinforced cement samples are estimated using the above fitted data. The relative peak area ratio (A_O-H_/A_H-C_) is estimated to be 0.386 for control cement, which decreases considerably to 0.289, when 1% jute is added to the cement. However, the addition of alkali treated jute fibre to the cement increases the relative peak area ratio to 0.358. Therefore, we observe that the raw jute reinforced cement sample results in less hydration of the cement product compared to that of the control cement sample. In comparison, the alkali treated jute cement sample produces more hydrated cement product compared to that of the raw jute cement sample. We observe that the addition of the raw jute fibre in cement delays the cement hydration reaction, which is counterbalanced by the addition of the alkali treated jute fibre. Interestingly, it is also observed from Table 3 that the relative peak area ratio of all three samples increases with increasing hydration time. Therefore, it is confirmed that the extent of hydration increases with increasing curing time. These effects can be further clarified by thermal analysis and free lime estimation.

### Thermal analyses (DSC and TG-DTG) confirm the formation of the extent of hydrated cement product

DSC thermograms of control, raw jute, and alkali treated jute fibre reinforced cement samples contain a number of endothermic peaks between 60–510°C (Fig. 2(e)). The peak at 60–130°C appears to be due to the loss of water from calcium silicate hydrate (C-S-H)^12^,^24^. The peaks in the temperature range 170–242°C and 345–430°C appear to be due to the decomposition of the monosulphate phase (C_3_A.CaSO_4_·12H_2_O) and hydrogarnet phase, respectively^12^. The characteristic endothermic peak ~ 443–510°C appears to be due to the decomposition of calcium hydroxide (Ca(OH)_2_). Estimating the area under the endothermic peak due to Ca(OH)_2_ decomposition, one can estimate the enthalpy change (ΔH) associated with the decomposition reaction. From Fig. 2(f), the enthalpy change value (ΔH) for control cement hydrated for 28 days is estimated to be 88.28 J/g, which is reduced to 72.05 J/g by the addition of raw jute in cement. However, the addition of the alkali treated jute fibre in cement increases the ΔH value to 78.57 J/g. Similar to the previous analyses, the enthalpy change value resulting from the decomposition of calcium hydroxide in DSC analysis of control, raw jute, and alkali treated jute cement samples increases gradually with the increase in curing time (Fig. 2(f)).

Figs. 2(g) and 2(h) show the TG and DTG plots of control, raw jute, and alkali treated jute reinforced cement samples hydrated for 28 days. As shown in Fig. 2(g), weight loss up to 200°C is attributed to the loss of either surface adsorbed water or water from the calcium silicate hydrate gel and ettringite^12^. The subsequent weight loss up to 400°C is attributed to the decomposition of various hydrated silicate and aluminate compounds. The characteristic weight loss at 420–444°C and 590–675°C appears to be due to the decomposition of Ca(OH)_2_ and calcium carbonate phases, respectively^25^. Usually, the higher mass loss due to the decomposition of the calcium hydroxide in the TG thermogram is indicative of more calcium hydroxide produced by the cement hydration reaction. The mass loss corresponding to the decomposition of calcium hydroxide for the raw jute cement sample hydrated for 28 days is 1.3% instead of 1.84% for the control sample. For the alkali treated jute cement sample, mass loss is estimated to be 1.42%. The same trend is also observed with DTG analysis (Fig. 2(h)). Therefore, adding raw jute to a cement system retards the cement hydration reaction. Adding alkali treated jute fibre to a cement system virtually eliminates the hydration retardation effect.

### Free lime content estimation quantitatively justifies the reduction of hydration delaying action

The free lime is liberated during the hydration of cement, and the amount of free lime is considered to be an indicator of the degree of cement hydration reaction^12^. Commonly, the free lime content is correlated with the cement hydration reaction. Fig. 2(i) represents the variation in the estimated free lime content as a function of the hydration time. From the figure, it is observed that the estimated free lime content for all the samples increases with increasing hydration time. Interestingly, as indicated in the figure, irrespective of the curing time, the raw jute reinforced cement sample shows less free lime content than the control and alkali treated jute reinforced cement sample counterpart. The alkali treated jute fibre reinforced cement sample shows a relatively higher amount of free lime than the raw jute reinforced cement sample.

## Discussion

**Figure 3:**
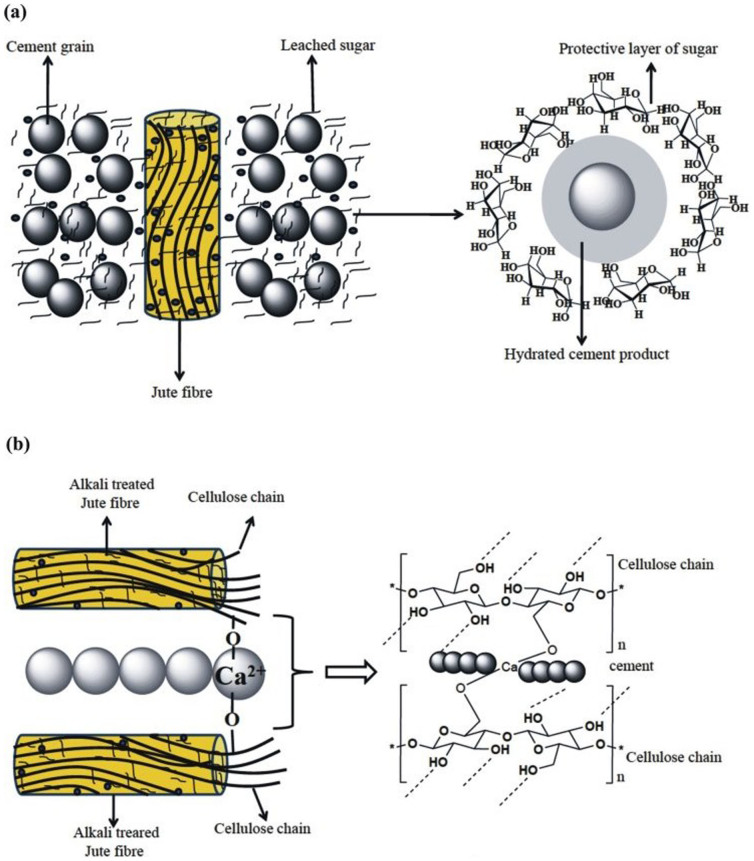
(a) Plausible mechanism for the formation of protective layer of leached sugar (from jute fibre) around the cement grain and (b) plausible mechanism for the formation of bonds between hydrated cement products with the cellulose chain of jute fibre.

**Figure 4:**
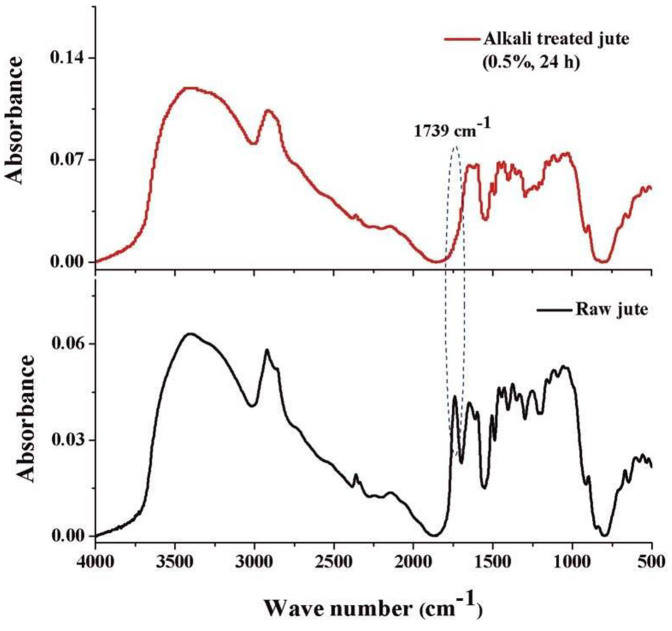
FTIR spectra of raw jute and alkali treated (0.5%, 24 h) jute fibre.

**Figure 5:**
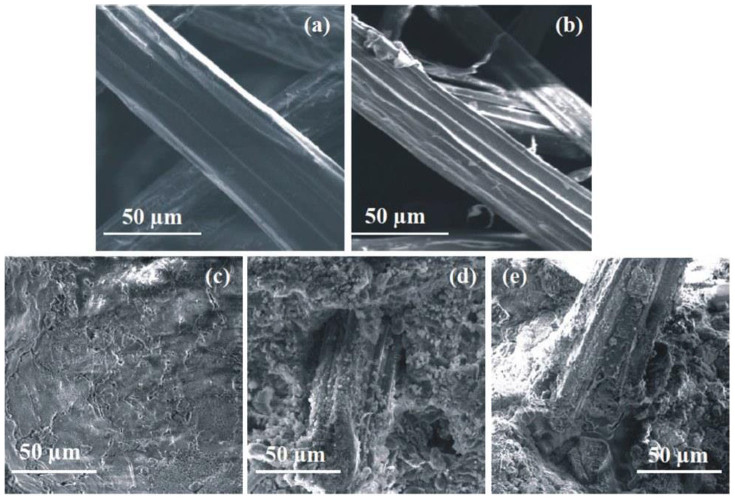
SEM images of raw and alkali treated jute fibres as well as raw and alkali treated jute fibre reinforced cement samples. (a) Raw jute fibre, (b) alkali treated (0.5%, 24 h) jute fibre, (c) control cement paste, (d) raw jute reinforced cement paste, and (e) alkali treated jute reinforced cement paste.

Raw jute fibre contains organic components such as hemicelluloses, lignin, pectin, and other compounds^18^. Accordingly, the jute fibre leaches loosely bonded organic compounds during mixing with a highly alkaline (pH ~ 12.76) cement matrix. These organic compounds are responsible for delaying the cement hydration reaction by either forming a protective layer around the partially reacted cement grain (Fig. 3(a)), or forming a chelate complex with the cations present in the hydrated cement^12^,^13^. Additionally, the chelation of the sugars with the cations present in hydrated cement may disturb the cement hydration reaction equilibrium and minimize the nucleation and growth of the hydrated cement product. However, alkali treatment removes the soluble sugars from the jute fibre^17^,^18^. This statement can be supported by FTIR analysis (Fig. 4) as well as SEM analysis (Figs. 5(a) and 5(b)) of the raw and alkali treated jute fibres. As indicated in Fig. 4, several peaks appear in the FTIR spectra of raw and alkali treated jute fibre due to the presence of the different functional groups reported by Roy et al.^18^. The most important characteristic peak appears at 1739 cm^−1^ for raw jute fibre and is absent for alkali treated jute fibre, indicating that the alkali treatment removes the loosely bonded hemicelluloses. It is reported that the absorption band at 1739 cm^−1^ appears to be due to the C = O stretching of the ester linkage in hemicelluloses^18^. Thus, from the FTIR analysis of the jute fibres, it is assumed that the alkali treatment is able to remove the loosely bonded soluble sugars from the jute fibre. Therefore, the mixing of the alkali treated jute fibre with the cement matrix reduces the possibility of the sugar leaching into the cement system, which minimizes the ion capturing possibility from the cement matrix. It is also reported by Roy et al.^18^ that the alkali treatment of jute fibre removes the soluble sugars from the fibre as well as increases the surface roughness, and alkali treatment splits the fibres into fibrils. Figs. 5(a) and 5(b) show SEM images of the raw as well as mild alkali (0.5%) treated jute fibres; the figures show that the surface roughness as well as the effective surface area of the jute fibre increase after alkali treatment. The increased surface roughness, as well as the fibrillation of the jute fibres due to alkali treatment, increase the effective surface area for bonding between the fibre and the matrix. Those factors may enhance fibre-matrix compatibility which improves the overall performances of alkali treated jute fibre reinforced cement composite. Figs. 5(c), 5(d) and 5(e) represent the SEM images of the fracture surface of control, raw jute and mild alkali treated jute reinforced cement samples hydrated for 28 days. In Fig. 5(c), adequate hairline cracks are clearly observed in the micrograph of the control cement paste. However, no such cracks are observed in the micrographs of the raw and alkali treated jute reinforced cement samples (Figs. 5(d) and 5(e), respectively. Additionally, a compact contact in between alkali treated jute fibres and the cement matrix apparent in the alkali treated jute cement sample (Fig. 5(e)) but not in the raw jute cement sample (Fig. 5(d)). Therefore, it can be concluded that the fibre matrix compatibility increases due to alkali treatment of the jute fibre. Moreover, based on the results, the alkali treated jute fibre reinforcement increases the cement hydration reaction product compared to that of the raw jute reinforced cement sample, but the alkali treated jute fibre reinforced cement sample still produces a less hydrated product than the control cement sample. This seems to be due to the bonding between internal cellulose chains of jute fibre with the cement matrix (Fig. 3(b)), which may enhance the fibre-matrix compatibility and enhances the strength of alkali treated jute fibre reinforced cement composite. Therefore, it is concluded that the mild alkali treated jute fibre reinforcement increases the fibre-cement compatibility as well as reduces the hydration delaying activity as compared to that of the raw jute fibre reinforcement. Additionally, the mild alkali treated jute fibre reinforcement would be an initial alternative approach to develop sustainable and green construction material.

## Methods

### Raw materials

In this investigation, the fly ash-based Portland-pozzolana cement (Ambuja cement) conforming to IS 1489–1991^26^, jute fibre (*Corchorus olitorius*; TD4 grade), and distilled water were used to prepare the control cement paste, raw jute reinforced cement paste, and alkali treated jute reinforced cement paste. The details of the raw materials used in this investigation are provided in the supplementary information. Additionally, the oxide composition of the cement is provided in Table S-1 (see supplementary information).

### Preparation of the hardened cement samples for the hydration analysis

To evaluate the effectiveness of alkali treated jute fibre on the hydration behaviour of cement by different analytical techniques, the hydrated control cement paste, raw jute reinforced cement paste, and alkali treated jute reinforced cement paste were prepared. In this investigation, the samples were prepared using a water to cement ratio (W/C) of 0.6. In the batch mixing of control, raw jute, and alkali treated jute reinforced cement samples, 500 g of cement was mixed with 300 ml of distilled water. Additionally, for the fabrication of the raw jute and alkali treated jute reinforced cement pastes, 5 g of saturated water was absorbed and 5 g of alkali treated jute fibres were used, respectively. Both the raw jute fibre and alkali treated jute fibre reinforced cement pastes were prepared using 1% (weight) jute fibre relative to the weight of the cement. In this investigation, prior to the fabrication of the raw jute reinforced and alkali treated jute reinforced cement pastes, the chopped jute fibres (TD4) were ground in a mixture grinder for homogeneous mixing with cement. Afterward, for the preparation of the alkali treated jute cement paste, the ground jute fibres were immersed in 0.5% NaOH solution for 24 h with a jute:NaOH solution ratio of 1:30. The excess liquor was then drained out after 24 h. The total amounts of the alkali treated wet jute fibre were then mixed with the total cement and water required to prepare the alkali treated jute cement paste, and mixed thoroughly for 5 min using a mechanical mixer. For the preparation of the raw jute reinforced cement paste, the ground jute fibres were immersed in distilled water for 24 h with a jute:water ratio of 1:30 to obtain saturated water absorbed jute fibre. After 24 h, the excess liquor was removed. Furthermore, the total amounts of saturated water absorbed jute fibres were used for the fabrication of the raw jute reinforced cement paste following the same procedure described above. Finally, the freshly prepared control, raw jute reinforced and alkali treated jute reinforced cement samples were kept in glass petri dishes for 24 h at ambient temperature (30 ± 2°C). The samples were then removed from the petri dishes and allowed to water cure for different curing times. A pictographic diagram for the fabrication of alkali treated jute cement paste is provided in Fig. S-1 (See supplementary information). In this study, the different analytical properties of the alkali treated jute (1%) reinforced cement sample (1AJC) were compared with the control cement (0CC) and the raw (1%) jute reinforced cement sample (1RJC). The formulation details of the control, raw jute reinforced and alkali treated jute reinforced cement samples are provided in Table S-2 (see supplementary information).

### Characterizations

To evaluate the effect of alkali treated jute fibre on the setting and the hydration behaviour of cement, setting time measurement, hydration test at an early age, and analytical characterizations of the control, raw jute reinforced, and alkali treated jute reinforced cement samples were performed. The details of the setting time measurement, hydration test and all of the analytical characterizations are provided in the supplementary information (see supplementary information).

## Author Contributions

S.C. wrote the main manuscript text and prepared the figures 1–5 and tables 1–3. B.W.J. has guided the research program. Both the authors have approved the final version of this manuscript. In this paper, both the authors (B.W.J. and S.C.) have contributed equally.

## Supplementary Material

Supplementary Information Supplementary Information

## Figures and Tables

**Table 1:** Standard consistency and setting times of control, raw jute, and alkali treated jute fibre reinforced cement samples

Sample code	Jute loading (%)	Standard consistency (%)	Initial setting time (min)	Final setting time (min)
0CC	0	35.0 ± 0.5	130 ± 07	170 ± 10
1RJC	1	36.5 ± 0.5	160 ± 09	205 ± 07
1AJC	1	36.5 ± 0.5	154 ± 09	194 ± 08

**Table 2:** Characteristic features of temperature vs. time plot (T_(max)_: maximum achievable temperature; t: time (minutes) required to reach maximum temperature; tr_(zero)_: time (minutes) required to reach zero rate of temperature change; tr_(max)_: time (minutes) required to reach the maximum rate of temperature change)

	Characteristic feature of temperature vs. time curve		Characteristic feature of temperature change rate vs. time curve	
Sample code	T_(max)_ (°C)	t (min)	tr_(zero)_ (min)	tr_(max)_ (min)
0CC	24.37	860	200	570
1RJC	24.05	1040	240	680
1AJC	24.07	1020	230	650

**Table 3:** XRD and FTIR analyses of control, raw jute, and alkali treated jute fibre reinforced cement samples hydrated for different times

			Peak area ratio for different hydration time			
Type of Analysis	Identity of peak areas	Sample code	7days	28 days	42 days	90 days
XRD	A_p_/A_a_	0CC	0.083	0.173	0.253	0.541
		1RJC	0.066	0.105	0.175	0.202
		1AJC	0.066	0.125	0.190	0.203
FTIR	A_O-H_/A_H-C_	0CC	0.226	0.385	0.473	0.766
		1RJC	0.136	0.290	0.386	0.431
		1AJC	0.170	0.359	0.430	0.440
