# Supplementary material for: A mild alkali treated jute fibre controlling the hydration behaviour of greener cement paste
Source: Sci Rep. 2015 Jan 16;5:7837. doi: 10.1038/srep07837 (PMC4296306; doi:10.1038/srep07837)
Supplement: Supplementary Information [file srep07837-s1.pdf]

**Supplementary Information**

# A mild alkali treated jute fibre controlling the hydration behaviour of greener cement paste

Byung-Wan Jo[1]* and Sumit Chakraborty[1]

[1]Department of Civil and Environmental engineering, Hanyang University, Seou, South Korea, 133791

## Methods

### Raw materials:

*Cement:* Fly ash based Portland-pozzolana cement (Ambuja cement) conforming with IS 1489-1[1] was used as the primary binding material for the preparation of the control, raw jute reinforced, and alkali treated jute reinforced cement pastes. The cement in this research was purchased from Ambuja Cements Ltd., India. The predominant oxide composition (analyzed) of this cement is shown in Table S-1.

**Table S-1:** The oxide composition of Portland pozzolanic cement

| Composition | $SiO_2$ | CaO | MgO | $Fe_2O_3$ | $Al_2O_3$ | C | Loss of ignition |
|---|---|---|---|---|---|---|---|
| Weight (%) | 27.28 | 50 | 1.96 | 6.18 | 9.20 | 0.76 | 2.66 |

*Jute fibre:* The jute fibre (*Corchorus olitorius;* grade TD4 (Tossa Desi) as per standard IS: 271[2] was used as a reinforcing agent in this research. The jute fibres were golden in color and about 2.5 m in length. They were collected from Gloster Jute Mill, Howrah, India. The received jute fibres were too long to be used for the fabrication of the jute reinforced cement paste; thus, to make the jute fibres usable they were chopped to ~ 5 mm.

*Corresponding author's information: joycon@hanmail.net , Ph: +82-2-2220-1804, Fax: +82-2-2292-0321

*Sodium hydroxide:* Sodium hydroxide (NaOH) (Merck, India) was used to modify the fibre surface.

**Preparation of the hardened cement samples for the hydration analysis:**

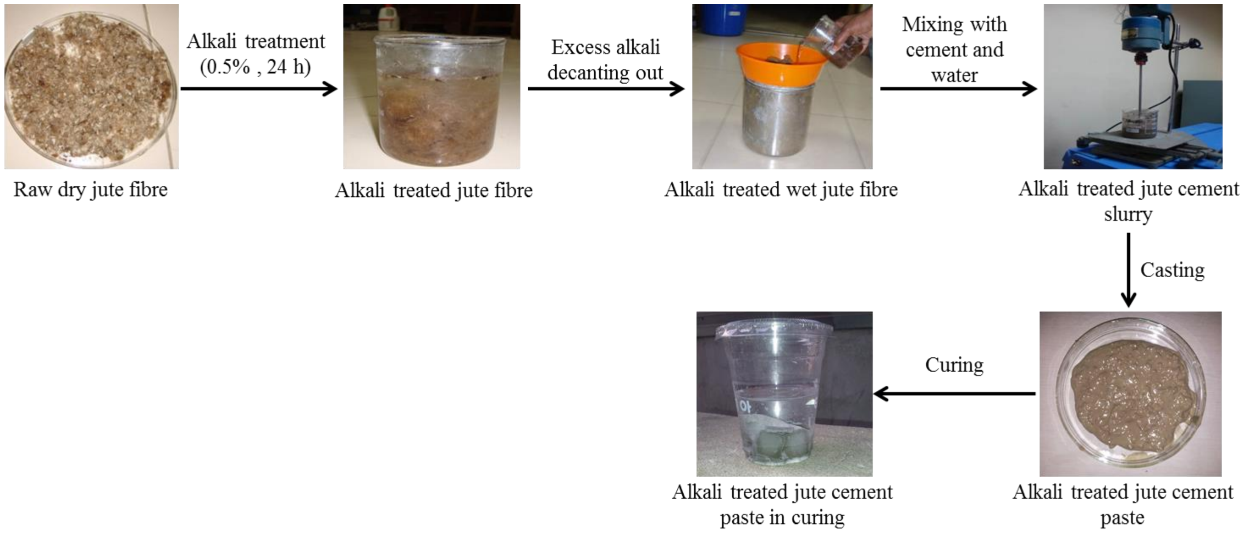


**Figure S-1** A pictographic diagram for the fabrication of alkali treated jute cement paste.

**Table S-2** The formulation details of the control, raw jute reinforced and alkali treated jute reinforced cement samples

| Type of cement sample | Components | | | | Sample code |
|---|---|---|---|---|---|
| | Cement (g) | Water (ml) | Raw jute (g) | Alkali treated jute (g) | |
| Control cement paste | 500 | 300 | -- | -- | 0CC |
| Raw jute cement paste | 500 | 300 | 5 | -- | 1RJC |
| Alkali treated jute cement paste | 500 | 300 | -- | 5 | 1AJC |

**Characterizations:**

To evaluate the effectiveness of the alkali treated jute fibre on the setting and hydration of cement, setting time measurement, early hydration test, and analytical characterizations were done.

*Standard consistency, setting time measurement*

The standard consistency and setting time of control, raw jute fibre reinforced, and alkali treated jute fibre reinforced cement pastes were measured using the Vicat apparatus in accordance with IS: 4031 part 4, 1988 and IS: 4031 part 5, 1988[3, 4].

*Early age hydration test*

The effect of alkali treated jute fibre on the hydration of the cement can be evaluated by the measurement of the evolved temperature during the cement hydration reaction. The test was performed using a water cement ratio (W/C) of 0.6. In a particular batch, 400 g cement was mixed with 240 ml of water in a polyethylene bag for the fabrication of the control cement paste. To fabricate the alkali treated jute cement sample, 4 g of ground jute was initially treated with 0.5% NaOH solution for 24 h followed by mixing with 400 g of cement and 240 ml of water. On the contrary, for the raw jute cement sample, cement and water were kept the same as the control sample. Four grams of saturated water absorbed jute fibre was used. Immediately after mixing, the tip of the temperature sensing thermocouple (Type J, purchased from URS, India) was inserted into the slurry. The bag was then placed in a Teflon thermo flask for 40 h and maintained at room temperature, 20°C. The connection of the thermocouple and the personal computer was made by a shielded cable. A text data format was generated by a data acquisition

card and LabVIEW 8.5 software (National Instruments, USA) followed by conversion of an analog signal to a digital one.

*Analytical characterization*

The X-ray diffraction pattern of the hydrated cement samples was recorded using an X-ray diffractometer (ULTIMA III, RIGAKU Inc. Japan). A monochromatic X-ray beam of wavelength 1.54 Å was obtained from the Cu $K_{\alpha}$ radiation and Ni filter. An XRD scan was performed using a scan speed of 1° $min^{-1}$ in 0.02" steps. For the measurement of the X-ray diffraction of the hydrated samples, the powdered specimens were packed in the rectangular hollow area of a glass sample holder.

Fourier transform infrared spectra (FTIR) of hydrated cement samples (without and with jute) were recorded using an FTIR spectrometer (Nexus 870, Thermo Nicolet Corp. USA). To record the FTIR spectra, 1 mg of the powder samples was mixed with 100 mg of KBr to make circular pellets. Thereafter, the spectra were recorded in the range of 4000 – 400 $cm^{-1}$ with 2 $cm^{-1}$ resolution and 32 scans.

Differential scanning calorimetry (DSC) of the hydrated cement samples was performed using a differential scanning calorimeter (DSC200PC, NETZSCH, USA). The DSC measurements were performed in the temperature range between 30–600°C maintaining a heating rate of 10°C $min^{-1}$ under a dynamic $N_2$ atmosphere. For effective comparison, the weights of the samples were kept identical (~ 10 mg) for all of the measurements.

A thermo gravimetric (TG) analyzer (TG209F1, NETZSCH, UK) was used for TG analysis of the hydrated cement samples. The weight of the samples was kept identical (~ 10 mg) for all of the TG measurements. The measurements were performed in a dynamic air atmosphere over a temperature range of 30 to 1000°C at a heating rate of 10°C $min^{-1}$.

SEM micrographs of the raw and alkali treated jute fibre as well as raw and alkali treated jute cement samples hydrated for 28 days were recorded using a scanning electron microscope (Vega-LSV, TESCAN, Czech Republic). To avoid charging of the nonconductive samples, a thin layer of gold was sputtered coated on the surface of the samples.

Finally, to evaluate the extent of the hydration products in the hydrated cement samples, the free lime content was estimated. The free lime content was estimated in accordance with the modified Frank extraction method [5]. To estimate the free lime content, 1 g of hydrated cement sample was refluxed with 40 ml of isopropyl alcohol and ethyl acetoacetate mixture (in 20:3 volume ratios) for 3 h. The refluxed solution was then cooled and filtered off using Whatman 41 filter paper. Finally, the filtrate was titrated against 0.1 N HCl using bromophenol blue as an indicator. The free lime content was estimated using the following relation.

$$\text{Percent of free lime} = 0.2804 \times \frac{V}{W} \ldots\ldots\ldots\ldots\ldots\ldots\ldots\ldots\ldots\ldots\ldots\ldots\ldots. (5)$$

where *v* is the volume of 0.1 N HCl required for titration and *w* is the weight of the cement sample.

**References**


[1] IS 1489-1. Portland-Pozzolana cement specification, part 1: Fly ash based [Third Revision] [CED 2: Cement and Concrete Sectional Committee] *Bureau of Indian Standards* (New Delhi, India, 1991, Reaffirmed 2005). Available at: https://archive.org/stream/gov.in.is.1489.1.1991/is.1489.1.1991#page/n17/mode/1up. Accessed: 19/11/2014.

[2] IS 271. Textiles — Grading of White, Tossa and Dalsee Uncut Indian Jute [Fourth Revision] [TX 03: Jute and Jute Products Sectional Committee] *Bureau of Indian Standards* (New Delhi, India, 2003). Available at: https://law.resource.org/pub/in/bis/S12/is.271.2003.pdf. Accessed: 19/11/2014.

[3] IS 4031-4. Methods of Physical Tests for Hydraulic Cement, Part 4: Determination of Consistency of Standard Cement Paste [First Revision] [CED 2: Cement and Concrete Sectional Committee] *Bureau of Indian Standards* (New Delhi, India, 1988, Reaffirmed 2005). Available at: https://archive.org/stream/gov.in.is.4031.4.1988/is.4031.4.1988#page/n2/mode/1up. Accessed: 19/11/2014.

[4] IS 4031-5. Methods of Physical Tests for Hydraulic Cement, Part 5: Determination of Initial and Final Setting Times [First Revision] [CED 2: Cement and Concrete Sectional Committee] *Bureau of Indian Standards* (New Delhi, India, 1988, Reaffirmed 2005). Available at: https://archive.org/stream/gov.in.is.4031.5.1988/is.4031.5.1988#page/n2/mode/1up. Accessed: 19/11/204.

[5] Presser, E. E., Brunauer, S., Kantro, D. L. & Weise, C. H. Determination of the Free Calcium Hydroxide Contents of Hydrated Portland Cements and Calcium Silicates. *Anal. Chem.* **33**, 877-882 (1961).
